# Editorial: Health inequities and reproductive justice in the modern era

**DOI:** 10.3389/frhs.2024.1414053

**Published:** 2024-05-01

**Authors:** Anna-Maria Aksan, Jennifer Schindler-Ruwisch

**Affiliations:** ^1^ Department of Economics, Fairfield University, Fairfield, CT, United States; ^2^ Department of Public Health, Fairfield University, Fairfield, CT, United States

**Keywords:** reproductive justice, gender equity, healthcare provider bias, sexual and reproductive autonomy, prenatal health

**Editorial on the Research Topic** Health inequities and reproductive justice in the modern era

The rise in reproductive injustice in the modern era continues to be alarming, disparately impacting the health and well-being of women and children globally. This collection of articles examines structural drivers of inequity as they relate to the core values of reproductive justice, the human right to maintain personal bodily autonomy, in particular a woman's autonomy over her sexuality and reproduction, and the ability to have and raise children in a safe and nurturing environment. A unifying conclusion from this collection of work is that the presence and impacts of bias and discrimination at the societal and provider level, including cultural norms surrounding a women's autonomy, racial disparities in the perinatal period, and harmful patterns of medical and health-provider training, demonstrate the importance of context-specific evaluation of interventions that promote reproductive and sexual health.

Chiziba et al. highlight the influence of power imbalance between sexual partners on contraceptive usage among married and partnered women in Zambia. Even when contraceptives are available, women are not always given shared decision-making in their use with their male partners. The modeling in this study sheds light on the variables that influence these partner dynamics and usage of contraception that may limit a woman's autonomy in these decisions. As Chiziba et al. concluded in their study, increased education did not always reflect equitable reproductive choice.

Likewise, this theme of equitable access of reproductive care to meet sexual health needs was portrayed in a differing geographical context in Maharashtra, India in an article by Shewale and Sahay. These authors closely examined the needs and behaviors of female sex workers in this high HIV prevalent region, illustrating the inadequacy of condom-focused interventions to protect against HIV/STI transmission as well as unintended pregnancies. Stigma, violence, discrimination, and gender inequity often disempower female sex workers from contraceptive use and reproductive choice. Although condoms can protect against HIV/STI transmission and unintended pregnancy, within contexts of sexual power imbalances, adherence is inconsistent and the need for non-barrier modes of contraceptives to prevent unintended pregnancy are an option to be further explored.

The role of social mobility and racial disparities were highlighted in the article by Hawkins et al. who analyzed high levels of perinatal depression among Black women in Detroit, Michigan. They illustrate that while economic status can be protective against depression, there is a surprising connection between upward (in addition to downward) social mobility over the life course with increased depression, contributing to a burgeoning literature on diminished returns to education and financial security experienced by historically marginalized groups in the United States. Rising incomes are not necessarily manifesting in the expected improved health outcomes when coupled with racism, discrimination and bias. The authors underscore the importance of improving equitable outcomes in clinical settings through medical training that emphasizes evidence-based screening, identification and support for depressive symptoms in pregnancy. In line with the implications highlighted above, education gaps among health care providers ranging from cultural insensitivity to clinical knowledge about abortion care, amplify inequities in health and reproductive outcomes.

Janušonytė et al. surveyed medical students across 85 countries and identified a gap in medical school preparation for abortion care specifically, and limited instruction on gynecology more generally. Given the broad willingness of those surveyed to provide abortion care, the authors attribute the training gap to a global lack of institutional support. A lack of skilled services can lead to less compassionate and effective care and can place women who are already in vulnerable positions at greater risk of adverse health outcomes. We see this in the experiences of the female sex workers in Maharashtra, India, interviewed by Shewale and Sahay. who face discrimination from healthcare providers when accessing antenatal care and abortion services, particularly in rural areas. Among these women, stigma, discrimination, violence and lack of knowledge and misinformation around pregnancy detection and contraceptive methods and efficacy were identified as barriers to access and effective usage of contraceptives.

Finally, in addition to social determinants of health, there are clear environmental injustices that limit maternal and child health. Outdated water infrastructure exposes large swathes of the US population to lead-contaminated water, with children and those in-utero most vulnerable to the potential consequences. The water crises of Flint Michigan and Washington DC were associated with reduced fertility rates and low birthweight outcomes. Kodjebacheva et al. surveyed women of reproductive age from the University of Michigan—Flint and found their knowledge of proper mitigation to be lacking, despite the vast media attention on the city's ongoing water crisis. They highlight improved interventions to protect pregnant women and children from lead exposure. This research also necessitates work to protect environments and conditions for women and children to thrive so that individuals do not need to take extraneous measures to receive basic needs, like water, safely.

Together this series spans international contexts to highlight the themes of reproductive injustice and health inequity for women and children, imploring clinicians, public health workers, and all those who interact with and support individuals during the critical reproductive years to reduce harmful practices that adversely impact their health and limit their rights.

